# Backward swimming in elongated‐bodied abyssal demersal fishes: Synaphobranchidae, Macrouridae, and Ophidiidae

**DOI:** 10.1111/jfb.16049

**Published:** 2025-01-08

**Authors:** Imants G. Priede, Alan J. Jamieson

**Affiliations:** ^1^ School of Biological Sciences, University of Aberdeen Aberdeen UK; ^2^ Minderoo‐UWA Deep‐Sea Research Centre, School of Biological Sciences and Oceans Institute, The University of Western Australia Perth Western Australia Australia

**Keywords:** abyssal, anguilliform, *Coryphaenoides*, deep sea, escape behavior, swimming

## Abstract

The deep‐sea demersal fish fauna is characterized by a prevalence of elongated‐body forms with long tapering tails. Using baited camera landers at depths of 4500–6300 m in the Pacific Ocean, we observed multiple instances of backward swimming using reverse undulation of the slender body in four species: the cutthroat eel *Ilyophis robinsae*, abyssal grenadier *Coryphaenoides yaquinae*, and cusk‐eels *Bassozetus* sp. and *Barathrites iris*. Backward swimming was used as an escape or repositioning maneuver, reversing for up to seven tail beats before resuming forward swimming in a new direction. The eel *I. robinsae* reversed with a swimming wave frequency of 0.51–0.95 Hz, wavelength 0.6–0.75 of the body length (*L*), and large amplitude movements of the head from side to side. *C. yaquinae* reversed relatively slowly at 0.21–0.52 Hz and wavelength 0.5–0.7 *L* aided by propulsive movements of the pectoral fins and minimal lateral movement of the head. The ophidiids also used reversed propulsive body waves augmented by paddling with the pectoral fins but with some lateral movement of the head. Pectoral‐fin movements in all species were in synchrony with the body movements. The elongated‐body form enables backward swimming by reversal of the anguilliform propulsive wave and has the advantage that the fish automatically returns to safety along the path recently traveled. This maneuverability conferred by an elongated body may be a significant factor in selection for body shape in deep‐sea fishes.

## 1 INTRODUCTION

The fundamental mode of locomotion in chordates is characterized by propagation of waves of lateral bending backward along the body (Di Santo et al., 2021). Several lower chordates, such as the lancelet (*Branchiostoma*), hagfishes (*Myxine glutinosa* L and *Eptatretus stoutii* [Lockington, 1878]) (Adam, 1960; Campbell, 1940), and lampreys (Islam & Zelenin, 2008), with an elongated body swim in this way and can also reverse the propulsive wave to swim backward. In early studies on *Branchiostoma*, before the advent of high‐speed cine‐photography, there was debate as to whether lancelets swim head or tail first (Arey, 1915); it is now well established that they can swim forward or backward with apparent equal facility (Webb, 1973). Ability to reverse the locomotor wave is embedded in the basic chordate motor nerve organization whereby the response of spinal motor neurons to signals from intraspinal stress receptor neurons is reversed by commands originating in the brain stem (Hsu et al., 2017). Videler (1993) observed that eels (Anguilliformes) are “experts in forward and backward swimming in mud and maze‐type environments.” D'Août and Aerts (1999) analysed the kinematics of forward and backward swimming in the European eel *Anguilla anguilla* L. and concluded that the elongated body shape with a relatively homogeneous muscle distribution along the body axis is conducive to the “reversed forward” mode of swimming.

In fishes that swim by undulation of the body, the amplitude of the propulsive wave is small in the anterior part of the body and increases toward the tail in so‐called body and caudal fin (BCF) propulsion (Blake 2004; Di Santo et al., 2021). In the evolutionary diversification of fishes, the trend for enhanced swimming performance has resulted in generally shorter body forms, with thrust mostly generated by the tail. This trend reaches its azimuth in the tunas with a high aspect ratio lunate tail, a narrow caudal peduncle, and muscle mass concentrated halfway along the body (Graham & Dickson, 2004). In sub‐carangiform, carangiform, and tunniform fishes, reversed BCF propulsion is ineffective or very inefficient. For example, the zebrafish (*Danio rerio*) retains the backward swimming nerve reflexes (Liao & Fetcho, 2008), but their activation is described as a “struggling” escape behavior (Deliagina et al., 2019) or as an aberrant behavior elicited by exposure to hallucinogenic drugs, such as lysergic acid diethylamide (LSD) (Kalueff et al., 2013). Reverse BCF propulsion requires large amplitude lateral bending of the anterior part of the body (D'Août & Aerts, 1999), which is not possible anatomically in short‐bodied fishes, although it has been demonstrated in some fish‐like swimming robots (Li et al., 2016).

Evolution of high‐performance swimming in fishes has, therefore, been at the expense of forward or backward maneuverability inherent in anguilliform locomotion. Use of median and paired fins for steering, braking, or propulsion can overcome this problem, and many neutrally buoyant acanthopterygian teleosts have abandoned BCF altogether (Webb, 1982) and utilize median and paired fin (MPF) propulsion for maneuvering in complex shallow‐water environments, such as coral reefs (Blake 2004; Friedman et al. 2021).

Webb (1982) points out that from the Palaeozoic onward there have been recurring evolutionary radiations toward elongate body forms in the Actinopterygii (ray‐fin fishes). Among deep‐sea demersal fishes, Neat and Campbell (2013) found a remarkable prevalence of elongated body forms with long tapering tails across multiple diverse taxa, including Chimaeriformes, Notacanthiformes (halosaurs and spiny eels), Anguilliformes (eels), Ateleopodiformes (jelly nose fishes), Aulopiformes (lizardfishes), Gadiformes (Cods, hakes, rattails), Ophidiiformes (pearlfishes, cusk‐eels), Scorpaeniformes (Liparidae, snailfishes), and Perciformes (Zoarcidae, eelpouts) (Priede, 2017), suggesting that the long tapering body form is strongly selected for (Martinez et al., 2021).

It has been assumed that in the darkness and relatively featureless environment of the abyssal plains, there is less need for an ability to navigate complex environments, so the simpler anguilliform body shape predominates (Martinez et al., 2021). However, many feeding opportunities for abyssal fishes occur in the form of food falls, such as dead cetaceans (Jones et al., 1998) or on complex topography of mid‐ocean ridges (King et al., 2006) continental slopes, sea mounts, and abundant abyssal rocky patches (Riehl et al., 2020). Abyssal fishes must therefore be able to navigate complex three‐dimensional environments without the benefit of visual cues but utilizing tactile and gustatory searching behavior (Wagner et al., 2007, Bailey et al., 2007). D'Août and Aerts (1999) suggested that backward undulatory swimming is particularly advantageous for species living in darkness as it allows the fish to escape hazards by reversing along a path recently traveled and tested. We hypothesize that for abyssal fishes, such escape ability is an important additional adaptive feature of the anguilliform swimming mode. Hitherto, there has been no evidence that deep‐sea fishes use such backward swimming.

**FIGURE 1:**
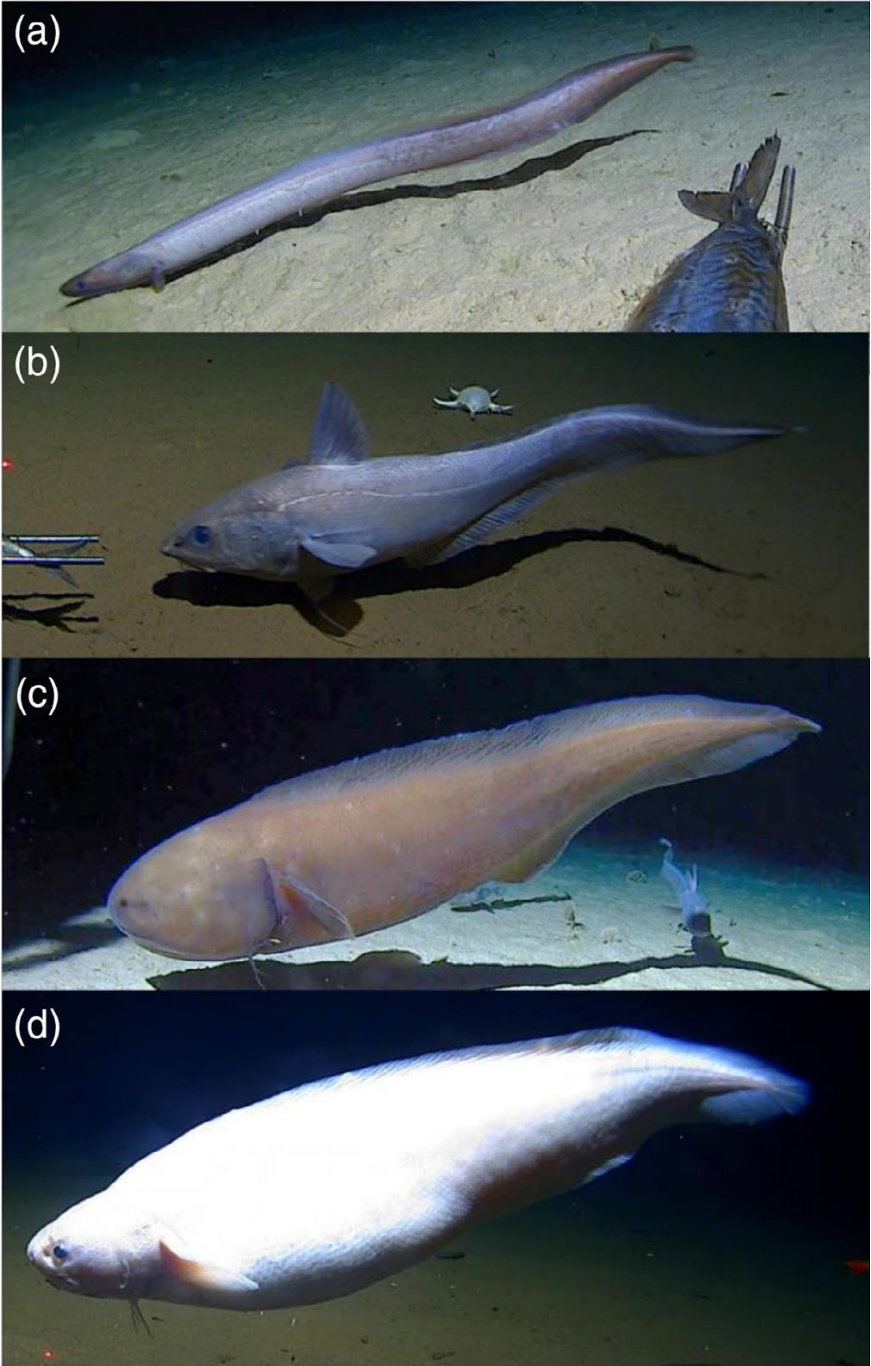
The four fish deep‐sea species observed swimming backward: (a) Synaphobranchidae (*Ilyophis robinsae*), (b) Macrouridae (*Coryphaenoides yaquinae*), (c) Ophididae (*Bassozetus* sp.), and (d) Ophidiidae (*Barathrites iris*). Note the elongated tapering body shape and the absent or minimal caudal fin.

Here we describe episodes of backward swimming by four different species of elongated‐bodied teleost fishes attracted to baited cameras on the abyssal floor of the Pacific Ocean, a cutthroat eel *Ilyophis robinsae* Sulak & Shcherbachev, 1997 (Synaphobranchidae, Anguilliformes), an abyssal grenadier *Coryphaenoides yaquinae* Iwamoto & Stein, 1974 (Macrouridae, Gadiformes), and two cusk‐eels *Bassozetus* sp. and *Barathrites iris* Zugmayer, 1911 (Ophidiidae, Ophidiiformes) (Figure 1) at depths of 4500–6300 m.

## 2 MATERIALS AND METHODS

### 2.1 Study sites

Data were collected from the research vessel (R.V.) *Dagon* during six legs of the Trans‐Pacific Transit Expedition (TPT) between Ensenada (Mexico), Hawaii (USA), and Tahiti (French Polynesia) from June 2, 2023, to January 12, 2024, as part of the Inkfish Open Ocean Program. A total of 123 baited camera lander deployments were completed at 43 sites along the 20,667 nautical mile ship's track. Additional data were obtained from 15 lander deployments during the Black Hole Expedition to the Sui Shin Hole in the center of the Philippine basin in July 2020 using the same vessel, then known as DSSV *Pressure Drop*. From the TPT expedition, 13 observations of backward swimming were obtained across the Polynesian region of the Central Pacific (33°N–5°S, 144°W–157°W) at depths of 4465–5568 m. One observation of backward swimming was made from the Black Hole Expedition (*ca*. 25°N 136°E) at 6328 m depth (Table 1).

**TABLE 1:** List of observations of backward swimming by abyssal fishes, with their locations and depths in the Pacific Ocean.

Ref	Station	Latitude°	Longitude°	Depth (m)	Family	Species	Period (s)	Hz	λ (L)
1	TP2_CR6_4500	−0.818	−144.303	4500	Synaphobranchidae	*Ilyophis robinsae*	1.44	0.69	0.6
2	TP2_MA6_4500	−0.8005	−144.299	4484	Synaphobranchidae	*I. robinsae*	1.05	0.95	0.7
3	TP2_OM7_4500	−3.9307	−144.032	4573	Synaphobranchidae	*I. robinsae*	‐	‐	0.75
4	TP3_OM5_4600	6.6435	−156.932	4816	Synaphobranchidae	Synaphobranchid	1.97	0.51	0.75
5	TP1_OM4_5600	32.0665	−148.769	5568	Macrouridae	*Coryphaenoides yaquinae*	1.914	0.52	≤ 0.5
6	TP2_CR7_4500	−3.925	−144.014	4588	Macrouridae	*C. yaquinae*	2.81	0.36	0.7
7	TP2_MA5_4900	4.4823	−145.883	4937	Macrouridae	*C. yaquinae*	3.46	0.29	0.5
8	TP4_OM2_5400	20.7042	−146.272	5445	Macrouridae	*C. yaquinae*	4.85	0.21	0.7
9	BH_CL4_6300	25.142	136.395	6328	Ophidiidae	*Bassozetus* sp.	5.55	0.18	≤ 0.5
10	TP2_CR7_4500	−3.925	−144.014	4588	Ophidiidae	*Bassozetus* sp.	‐	‐	≤ 0.5
11	TP2_OM5_4900	4.4873	−145.866	4944	Ophidiidae	*Bassozetus* sp.	11	0.09	‐
12	TP6_CR5_4500	−4.6162	−146.773	4465	Ophidiidae	*Bassozetus* sp.	2.26	0.44	≤ 0.5
13	TP2_CR6_4500	−0.818	−144.303	4500	Ophidiidae	*Barathrites iris*	3.27	0.31	0.7
14	TP2_CR6_4500	−0.818	−144.303	4500	Ophidiidae	*B. iris*	1.98	0.5	0.6

*Note*: Period, Hz, and λ refer to the observed backward swimming wave, where λ = wavelength expressed in terms of body length *L*.

Abbreviations: Ref, reference number of the video; station, lander deployment code.

### 2.2 Equipment

Three baited camera landers were deployed at each site in a 2‐km‐wide triangle. They descended to the seafloor by virtue of a ballast weight, a single steel cube of 126 kg, that was jettisoned at the end of the mission. Once the ballast was released, the lander returned to the surface through uplift from positively buoyant syntactic foam (TG39/11,500, Trelleborg Applied Technologies, Boston, MA, USA).

Each lander was equipped with a high‐definition (HD) video camera (IP Multi SeaCam 3105; Deep Sea Power and Light, San Diego, CA, USA) facing horizontally along a 1‐m‐long arm baited with one mackerel (*Scomber* sp.) carcass resting on the seafloor. Illumination was provided by a single diffused LED lamp (LED SeaLite). Conductivity, temperature, and depth (CTD) sensors (SBE49 FastCAT, Seabird Electronics, Bellevue, WA, USA) were mounted on the landers and connected to bespoke control systems. All lander systems were powered by three 24‐V lead acid batteries (SeaBattery). Each lander remained on the seafloor for ~6–8 h.

### 2.3 Species identification

No voucher specimens were caught during these expeditions; therefore, species identification was derived only from video (Figure 1). The synaphobranchid was identified as *I. robinsae*, based on the tapering snout and distribution of pores on the head (Sulak & Shcherbachev, 1997). For *C. yaquinae* and *B. iris*, the identifications are confident, as they are well‐documented species from these areas. However, *Bassozetus* is a highly cryptic genus in which multiple morphotypes are known, and therefore we assigned it to *Bassozetus* sp., putatively morphotype 1, described in Jamieson et al. (2021), with a long head, rather pointed snout, almost straight profile, and dorsal‐fin origin behind the operculum.

### 2.4 Image analysis

All video imagery from the seafloor was interrogated using EventMeasure software (SeaGIS; version 6.42). All visible mobile fauna were digitally annotated and identified to the lowest taxonomic resolution possible. Sequences that included backward swimming were identified manually and extracted for analysis (Table 1). Each sequence was replayed frame by frame, and an unobstructed complete cycle of body oscillation was identified. Period (*t*) and frequency (*f* = *1/t*) were measured, and wavelength (*λ*) was expressed as a fraction of body length (*L*). *t* and *f* were determined within the 40‐ms resolution of the 25‐Hz frame rate of the camera, estimates of *λ* were approximate, as the fish orientation was generally not normal to the axis of the camera. A series of approximately 12 still images, taken at regular intervals during a representative single cycle of swimming motion for each species, was captured for detailed analysis.

### 2.5 Ethics statement

The interactions with and observations of wild animals complied with the Programme in Animal Welfare, Ethics and Science (PAWES) as approved by the University of Western Australia, protocol identifier: 2022/ET000055.

## 3 RESULTS

### 3.1 Synaphobranchidae: *I. robinsae*

**FIGURE 2:**
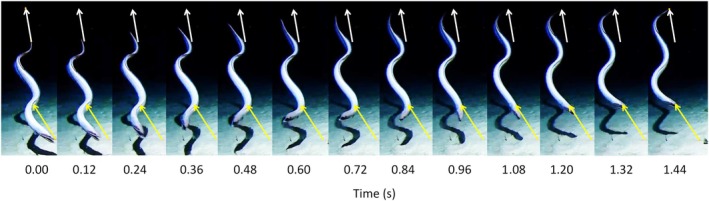
Time‐lapse sequence of a single cycle of backward swimming by an eel, *Ilyophis robinsae*, at 4500 m depth beneath the tropical central Pacific Ocean, 0.818°S, 144.303°W, ascending backward off the seafloor. The arrows indicate the direction of movement and connect the initial and final positions of the tip of the nose and tail (station: 1 TP2_CR6_4500).

Four examples of backward swimming were observed (Table 1; Figure 2). At deployment 1, the eel was feeding on the seafloor with its head beneath the bait support arm of the lander whereupon it escaped backward over approximately two body lengths (Video S1). It then turned to its left by bending the body and swam forward away from the lander. Figure 2 shows one cycle of the backward swimming wave propagating forward from the tail. The head oscillates from side to side over a large amplitude as exemplified by comparison of the image at 0.60 s with the start (0.00 s) and end (1.44 s) positions. At deployment 2, an individual reversed off the seafloor near the lander for four tail beats before bending its body toward the left to swim forward away from the lander (Video S2). At deployment 3, after attempting to feed on the bait, the eel swam backward, ascending out of view of the camera (Video S3). Possibly, a second species was observed at deployment 4, feeding at the bait and then departing backward for approximately two tail beats before turning by bending its body in a C shape to the left and swimming away in a forward direction (Video S4).

Except in deployment 3, where the fish moved out of the field of view, the backward swimming was a short escape or departure maneuver over about two body lengths before the individual turned to swim forward away from the lander. With *λ* values of approximately 0.7, a complete propulsive wave was evident within the length of the fish.

### 3.2 Macrouridae: *C. yaquinae*

**FIGURE 3:**
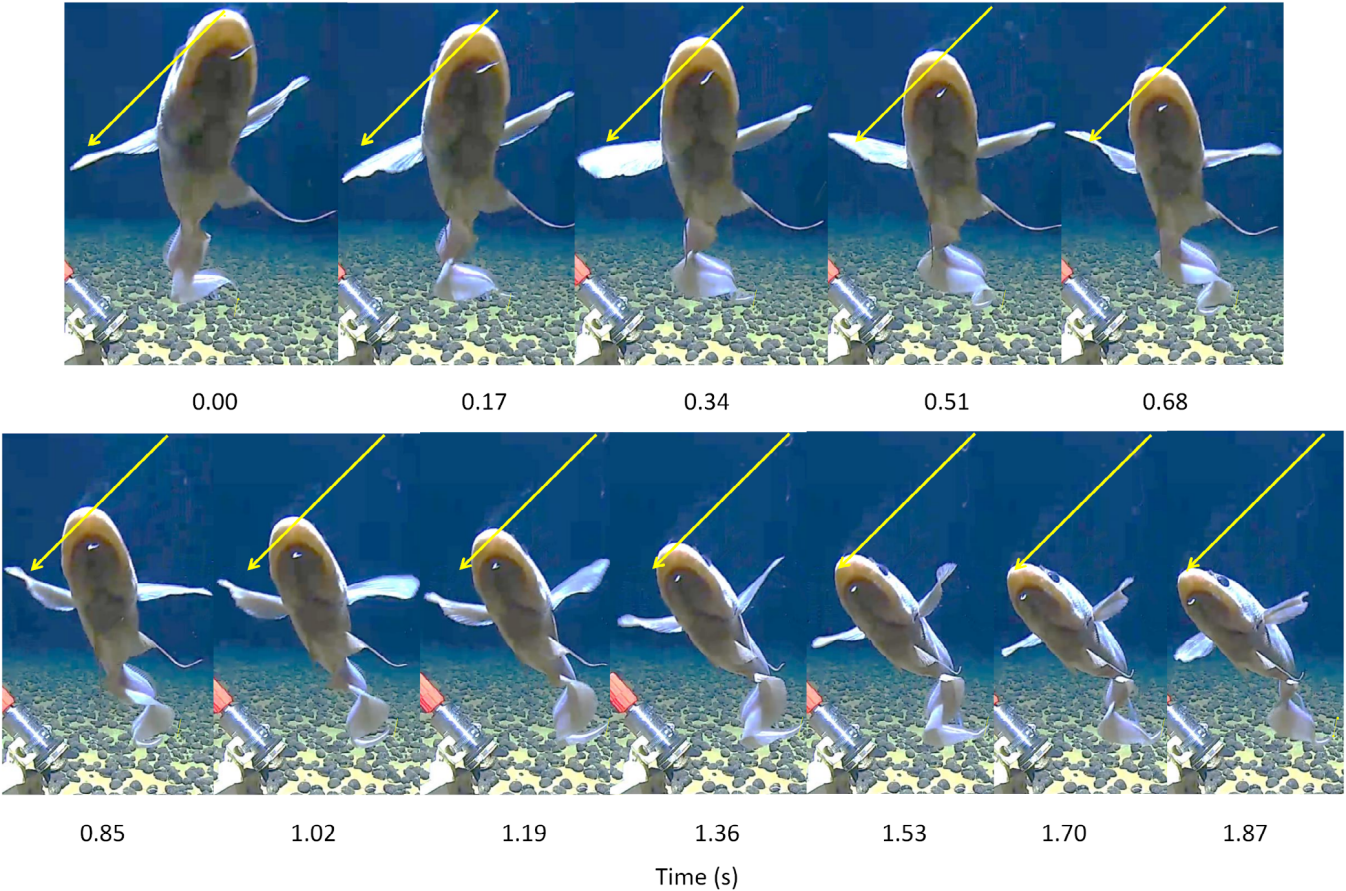
Time‐lapse sequence of a single cycle of backward swimming by an abyssal grenadier or rattail, *Coryphaenoides yaquinae*, at 5568 m depth beneath the central North Pacific Ocean, 32.0665°N, 148.769°W, reversing away from the lander camera. The arrows indicate the direction of movement and connect the initial and final positions of the tip of the nose (station: 5 TP1_OM4_5600).

Four examples of backward swimming by *C. yaquinae* were observed. At deployment 5 (Figure 3) the nose of the fish was above the camera, and it swam backward for five tail beats, presenting a view of its ventral surface to the camera as it moved away into the center of the field of view (Video S5). The pectoral fins executed a backward paddling motion, where the left and right fins were 180° out of phase with one another but in synchrony with tail beats (Figure 3). In contrast to the eel *I. robinsae* the head did not oscillate from side to side; in Figure 3, the tip of the nose follows the line marking the straight track between the start and end positions. After the backward escape maneuver, the fish turned to its right by bending the body in an arc and aided by a power stroke of the left pectoral fin continued swimming forward away from the lander.

At deployment 6, the fish was below the camera and moved backward using both reverse body undulations and pectoral‐fin motions, presenting its dorsal surface to the camera. Having completed the move away from the camera, it then began forward swimming toward the camera but used its pectoral fins as hydroplanes to ascend and pass safely above the camera (Video S6). At deployment 7, the fish was trapped, upside down on the sediment beneath the bait retaining bars of the lander. It extricated itself by backward swimming with combined reverse body undulations and pectoral‐fin paddling at the sediment interface. It then righted itself by bending the body in a tight “C” shape and, aided by movement of the pectoral fins, turned and straightened its body to swim forward away from the lander (Video S7). At deployment 8, the fish was observed moving slowly backward aided by the bottom current passing waves along its body and paddling with the pectoral fins. After this backward excursion, it began swimming forward against the current (Video S8). In all four cases, the macrourid used a reversed propulsive wave of the body and tail together with alternating paddling motions of the pectoral fins to swim backward away from the lander.

### 3.3 Ophidiidae: *Bassozetus* sp.

**FIGURE 4:**
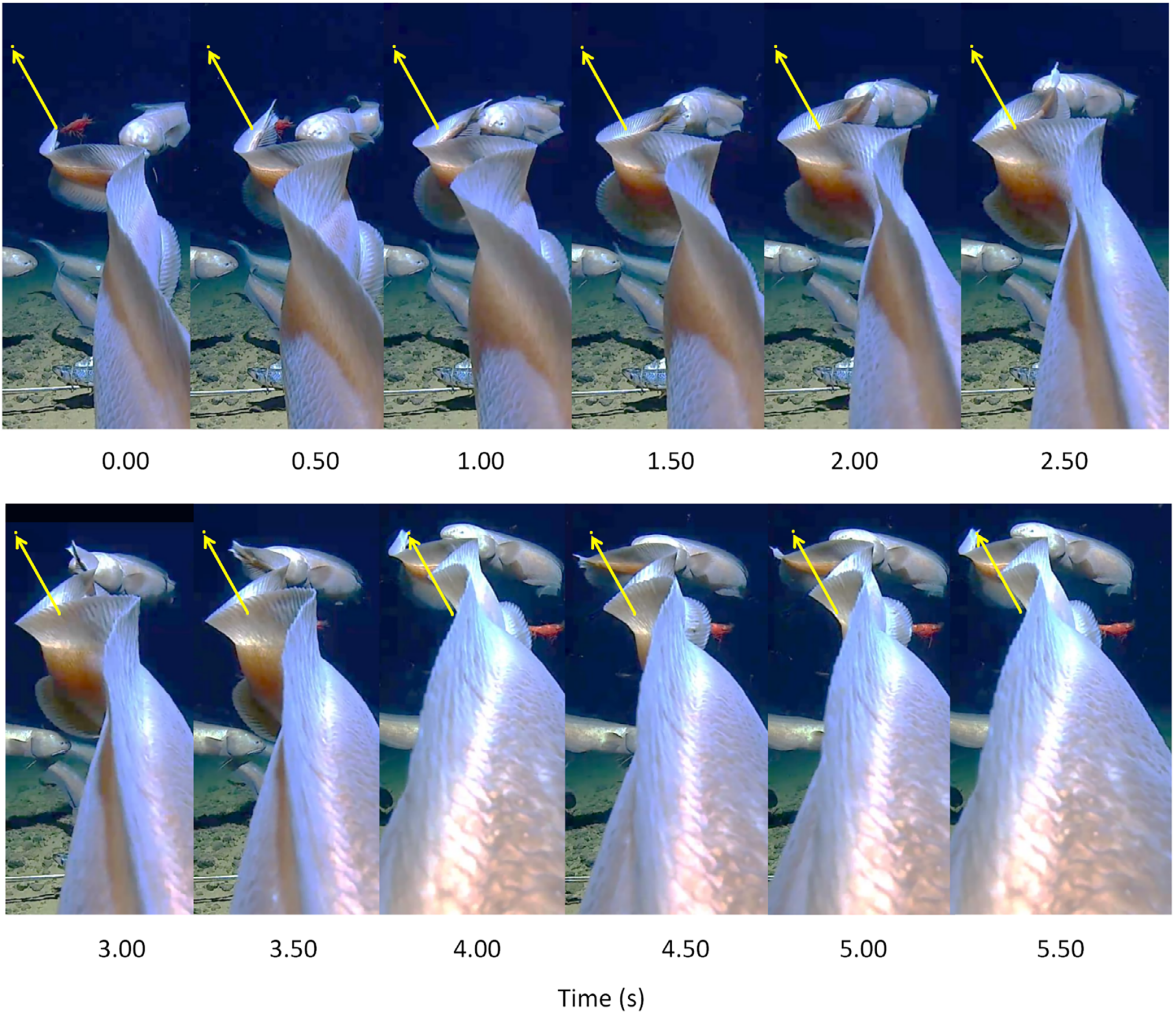
Time‐lapse sequence of a single cycle of backward swimming by a cusk‐eel, *Bassozetus* sp., at 6328 m depth in the Sui Shin Hole, Philippine basin, 25.142°N, 136.395°E, reversing away from the lander camera. Other individuals of the same species are in the background. The arrows indicate the direction of movement and connect the initial and final position of the tip of the tail (station: 9 BH_CL4_6300).

Four examples of backward swimming by the cusk‐eel *Bassozetus* were recorded. At deployment 9, the fish was beneath the camera and retreated swimming backward into the center of the field of view (Video S9). The dorsal view along the body shows propagation of the propulsive wave forward along the body (Figure 4). Synchronous paddling of the pectoral fins alternating side to side could be seen when the whole fish came into view. Once it had retreated, it resumed forward swimming in a slightly different direction, avoiding the camera.

At deployment 10, the fish encountered the camera on its left side and swam backward away from the camera before turning by bending the body to swim forward in a new direction (Video S10). At deployment 11, the fish was close below the camera, moving backward with the aid of slow pectoral fin paddling before turning by bending the body (Video S11). At deployment 12, the fish was above the camera, and backward swimming motion was clear in the shadow cast on the seafloor. After seven tail beats, the fish came into the field of view and then bent the body to the left to swim forward in a new direction. The pectoral fins paddled alternating side to side in synchrony with the body propulsive wave (Video S12).

### 3.4 Ophidiidae: *B. iris*

**FIGURE 5:**
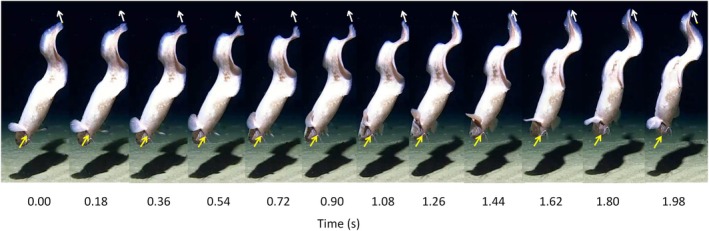
Time‐lapse sequence of a single cycle of backward swimming by a cusk‐eel, *Barathrites iris*, at 4500 m beneath the tropical central Pacific Ocean, 0.818°S, 144.303°W, ascending backward off the seafloor. The arrows indicate the direction of movement and connect the initial and final positions of the tip of the nose and tail (station: 14 TP2_CR6_4500).

Two instances of backward swimming were recorded. At deployment 13, the nose of the fish was under the bait from where it retreated by swimming backward for three tail beats, with synchronous movements of the pectoral fins before swimming forward after it had avoided the obstruction (Video S13). At deployment 14, the fish swam backward, ascending from the seafloor using pectoral fin sculling and passing waves along the body (Figure 5) for three tail beats (Video S14). Having completed its ascent, the fish resumed forward swimming. At 1.62 s (Figure 5) or 4 s into Video S14, there was a possible suction feeding event, with movements of the jaws and opercular apparatus.

### 3.5 Fish size, speed, and Reynold's number

All the fish observed were of the similar size, in the range 0.3–0.6 m overall length (*L*). Speeds were 0.02–0.1 m ∙ s^−1^. Assuming a kinematic viscosity (*v*) of 1.8 × 10^−6^ m^2^ ∙ s^−1^ for seawater at approximately 2°C on the seafloor, this gives a range of values of Reynold's number ($R_{e}=\frac{\mathrm{uL}}{v}$) for the observed swimming behavior of 6 × 10^3^ to 6 × 10^4^.

## 4 DISCUSSION

Backward swimming was observed in three deep‐sea demersal fish families: Synaphobranchidae, Macrouridae, and Ophidiidae. In all instances, a wave of lateral bending of the body propagated from the tail toward the head, which is a reversal of the normal propulsive wave used for forward swimming. These behaviors were in response to encounters with obstacles on the seafloor, such as the frame of lander, camera, or associated hardware. The backward swimming was sustained for a maximum of seven tail beats before individuals changed direction to resume swimming headfirst. The question arises as to whether such encounters with hardware of the lander represent normal behavior. Jones et al. (1998) observed the macrourid *Coryphaenoides armatus*, ophidiid *Spectrunculus grandis* (Günther, 1877), and zoarcids *Pachychara bulbiceps* (Garman, 1899) feeding at a dolphin carcass in the abyssal North Atlantic Ocean, consuming soft tissue, navigating the carcass, capturing small prey items while moving around protruding ribs, skull, and other skeletal elements. Cetacean falls are an important feature of the deep‐sea floor, supporting a specialized fauna and attracting mobile opportunistic scavengers (Smith et al., 2015). Macrourids (Linley et al., 2013) and ophidiids were also recorded on the rough terrain of the Mid‐Atlantic Ridge (King et al., 2006).

In the present study, the abyssal eel *I. robinsae* displayed a large‐amplitude swimming wave along the entire length of the body, very similar to that described by D'Août and Aerts (1999) for reverse swimming of *A*. *anguilla* (Figure 2). During backward swimming, the amplitude of the propulsive wave is rather uniform along the body, with large amplitude movements of the head in contrast to forward swimming, in which amplitude increases toward the tail (Di Santo et al., 2021). The tail beat frequency during backward swimming, 0.51–0.95 Hz, was similar to values of 0.6–1.2 Hz recorded for routine forward swimming in the deep‐sea cutthroat eel, *Synaphobranchus kaupii*, Johnson, 1862 at 500–2000 m depth on the continental slope of the Northeast Atlantic (Bailey et al., 2005).

The macrourid *C. yaquinae* also reversed the body propulsive wave along the body. However, backward swimming was slow with a mean tail beat frequency of 0.34 Hz (Table 1) compared to a mean of 0.73 Hz during forward swimming in this species in the abyssal (0.73 Hz) and hadal Pacific Ocean (0.68 Hz) (Priede et al., 2003; Priede et al., 2024). This aligns with the observations by Islam and Zelenin (2008) that backward swimming in the lamprey is generally slow. During backward swimming, the head did not yaw from side to side (Figure 3), as observed by D'Août and Aerts (1999) in *A. anguilla* or *I. robinsae* in the present study. In addition to the body propulsive wave, *C. yaquinae* used backward paddling of the pectoral fins, which were synchronized with the body swimming waves. In view of the relatively large size of the pectoral fins, they probably make a significant contribution to backward thrust. In one case (deployment 10, Table 1, Video S10), the fish used reverse swimming to escape from entrapment between the bait and the seafloor.

In the ophidiid *Bazzozetus* sp., the backward swimming resembled that observed in *C. yaquinae*, using a combination or a large‐amplitude reverse body wave and paddling of the pectoral fins. The tail beat frequency was slow (0.09–0.44 Hz), and the wavelength was short, with a complete swimming wave in the posterior region of the body. *B. iris* showed a distinct large‐amplitude reversed propulsive swimming wave, mainly confined to the caudal region (Figure 5), combined with reverse paddling of the pectoral fins. The blade of the pectoral fin was vertical during the power stroke and feathered during the backward recovery stroke.

Pectoral fins can play an important role in steady swimming and maneuvering behavior of fishes (Drucker et al., 2005). In the present study, the small pectoral fins in the eel *I. robinsae* do not appear to make a significant contribution toward backward swimming. In the macrourid, *C. yaquinae*, the pectoral fin was clearly active with alternating left and right oscillation. During normal forward swimming in this species, the blades of the pectoral fin are rotated to the horizontal plane (see video in Priede et al., 2024), apparently acting as hydroplanes regulating height above the seafloor. During braking and turning maneuvers, the fin blades are turned to vertical orientation, as observed during the forward power stroke of backward swimming but were folded or feathered during the recovery stroke (Figure 3).

There was a similar rowing action of the pectoral fins in the ophidiids during backward swimming, always synchronized with the body wave. The ophidiids apparently could not rotate the pectoral fins entirely to the horizontal plane as we observed in *C. yaquinae*. In both macrourids and ophidiids, the pectoral‐fin movements were always in synchrony with the body swimming waves. In the eel *I. robinsae* the head was seen to yaw from side to side during backward swimming, a phenomenon also observed in *A. anguilla* by D'Août and Aerts (1999), in contrast to forward swimming, in which the head moves through a very small amplitude (Di Santo et al., 2021). During backward swimming in the macrourids, side‐to‐side oscillation of the head was absent and somewhat reduced in the ophidiids, probably owing to action of the pectoral fins.

After the backward escape maneuver, the fish would usually turn before swimming forward in a new direction. The turn was initiated by bending the body in a “C” shape, so that the head was pointing toward the new direction of travel. In the eel *I. robinsae* pectoral fins did move in synchrony with the turn but did not seem important for the maneuver. In the macrourid *C. yaquinae* the turn was initiated by bending of the body, but a clear backward power stroke from the pectoral fin ensured a sharp turn toward the new direction. In the ophidiids, *Bassozetus*, and *B. iris*, the pectoral fins seemed less important in executing any turn. In none of the maneuvers observed were the pectoral fins seen to move independently or out of phase with the movements of the body, suggesting that the pectoral‐fin movements may be driven by the same locomotor neuronal networks that control the main body swimming wave (Deliagina et al., 2019).

Ability to reverse the movements of forward locomotion appears to be a universal feature of chordates from agnatha (Islam et al., 2006) to mammalia (Harnie et al., 2021) and is controlled by neural sensorimotor circuits within the spinal cord. McClellan (1989) observed that sea lampreys (*Petromyzon marinus*) encountering an obstacle can retreat by complete reversal of the swimming undulations of the body. Islam et al. (2006); Islam and Zelenin (2008) recognized such short bouts of slow backward swimming as an escape behavior and showed that in the river lamprey (*Lampetra fluviatilis*) longer episodes can be elicited by tactile stimulation of a large area of the anterior part of the body. Islam and Zelenin (2008) point out that in the lamprey, natural episodes of backward swimming are short and infrequent, in agreement with our observations for these deep‐sea teleosts. Lampreys have been studied in greatest detail, but backward swimming reflexes have also been described in the laboratory preparations of the spiny dogfish (*Squalus acanthias* L.) (Grillner, 1974). There is no evidence in nature that any Chondrichthyes can swim backward in this way; reverse body wave swimming is most likely in deep‐sea Hexanchiformes and Squaliformes that do not have a high aspect ratio caudal fin. Duration of backward swimming is likely limited by three factors: sensory, respiratory, and efficiency. From the sensory aspect, backward swimming negates the advantages of cephalization, where sense organs are primarily directed in the forward direction; as a result, searching behavior and avoidance of hazards would be impaired. During backward swimming, normal irrigation of the gill may be reduced owing to reversal of water flow over the head. Studies on eels (D'Août & Aerts, 1999) and robotic fishes (Li et al., 2016) show that backward BCF swimming is not as efficient as swimming in the forward direction.

The bottom currents in the abyssal Pacific Ocean have a tidal periodicity with a peak velocity of 0.05 m ∙ s^−1^ (Connolly et al., 2020; Wilson & Smith, 1984). Fish searching for food sources on the seafloor typically swim independently of water flow until an odor plume is encountered, where they turn toward the odor source and arrive at bait swimming against the current (Priede et al., 1991). The Reynold's number (Re) for forward or backward swimming is in the range 6 × 10^3^ to 6 × 10^4^, which is just above the critical Re≃3 × 10^3^ derived for anguilliform swimming (Gazzola et al., 2014). The deep‐sea species are swimming with some of the lowest Re values recorded for fishes but are nevertheless in the turbulent flow regime with inertial thrust generation (Videler, 1993).

Three hypotheses have been advanced to explain the prevalence of elongated‐body forms in abyssal demersal fishes: sensory, energetics, and maneuverability. Wynne‐Edwards (1962) first proposed the sensory hypothesis, suggesting that the elongated body helps increase the length of the lateral‐line sense organ improving its sensitivity and spatial resolution for detection of prey and other features in the darkness of the abyss (Priede, 2017). The lateral‐line canals in *Chimaera* extend to the tip of the long whip‐like tail (Wynne‐Edwards, 1962) and, also, along the exceptionally long snout of the Rhinochimaeridae (long‐nosed chimaeras). Chimaeras generally swim by flapping of the pectoral fins (Combes & Daniel, 2001) while the axis of the body remains straight, further maximizing the sensitivity of the lateral‐line sense organs. An interesting analogous case is the gymnotiform electric fish, *Apteronotus albifrons*, that also swims with a rigid body, as it scans the environment propelled by undulations of the anal fin. The maximum sensitivity of a linear sensory array, such as the gymnotiform electroceptors, is at the midpoint of the array, perpendicular to its axis. Therefore, small prey tend to be detected after the predator has moved past the target. It must then retrace its path to capture the prey, a special case of backward swimming behavior (Lannoo & Lannoo, 1993). The sequence in Figure 5 may be evidence of such a foraging reverse maneuver in the deep sea, with a putative suction feeding event by B. *iris* while swimming backward above the seafloor. These abyssal species do not swim with a rigid body, but this does not preclude alternating periods of drifting on the bottom current to enhance lateral‐line sensitivity alternating with active swimming. Importance of the lateral line is indicated by enlarged hindbrain areas in the abyssal grenadier *C. armatus* (a close relative of *C. yaquinae*), important for processing of information from the acoustic‐lateralis system (Priede et al., 1999; Wagner, 2001).

The energetic hypothesis stems from the observation that the anguilliform mode of swimming by undulation of a long, slender body is extraordinarily energetically efficient at slow speeds, enabling long‐distance transocean migrations by eels (*A. anguilla*) at minimal cost (van Ginneken et al., 2005). Several abyssal demersal fish species such as *Coryphaenoides* spp. are globally distributed (Gaither et al., 2016) and are continuously moving in their search for food, possibly traveling long distances across the seafloor, with some evidence for seasonal migrations on a transocean basin scale of thousands of kilometers (Milligan et al., 2020; Priede et al., 1991, 2003). Traveling such distances in the extreme oligotrophic conditions of the abyss has probably favored elongated bodies for locomotor efficiency (Martinez et al., 2021; Neat & Campbell, 2013).

The maneuverability hypothesis arises from D'Août and Aerts (1999), who state that the long, slender body facilitates backward undulatory swimming. Neat and Campbell (2013) further proposed that the presence of continuous dorsal and ventral fins along the elongated body facilitates backward swimming and rapid braking for fish deep‐sea fish living in darkness. Shallow‐water reef‐dwelling species tend to have laterally compressed, short, deep body shapes that confer great maneuverability in three dimensions mediated by visual interactions in the sunlit environment (Martinez et al., 2021; Miller et al., 2022). In the darkness of the abyss, anguilliform backward swimming has the great advantage that the fish automatically reverses along the presumed safe path recently traveled.

## 5 CONCLUSIONS

D'Août and Aerts (1999) comment that backward undulatory swimming seems highly functional for species living in darkness, enabling them to reverse along the path already traveled. They point out that only fishes with elongated‐body form use this form of locomotion, including eels, morays, congers, lampreys, and the eel catfish (*Channallabes apus* [Günther, 1873]). Hagfishes, *M. glutinosa* and *E. stoutii*, can also swim backward (Adam, 1960; Campbell, 1940). Here we show that some deep‐sea demersal anguilliforms, macrourids, and ophidiids can also swim backward. The backward swimming motions of the deep‐sea eel, *I. robinsae*, are the same as observed in the European eel *A. anguilla* and probably in most eels. The macrourids and ophidiids also swam backward by reversing the direction of undulation of the body, but the pectoral fins were also active. We conclude that in these species the pectoral fins contribute to thrust during backward swimming and can eliminate the large‐amplitude lateral movements of the head that is characteristic of eels when they swim backward. The pectoral fins seem incapable of working out of synchrony with the body swimming movements.

The elongated‐body form of deep‐sea demersal fishes confers enhanced maneuverability that may be important when foraging on complex bottom terrain or large carrion falls on the seafloor. The predominance of elongated‐body forms in deep‐sea demersal fishes is likely the result of the additive effect of several factors, including sensory, energetic, and maneuverability considerations. The anguilliform or elongated‐body form is highly versatile and has appeared on multiple occasions in adaptive radiation of the Actinopterygii (Webb, 1982), including the deep sea (Neat & Campbell, 2013).

## AUTHOR CONTRIBUTIONS

Conception of the study and manuscript preparation—Imants G. Priede and Alan J. Jamieson. Funding and collection of data—Alan J. Jamieson. Data analysis—Imants G. Priede.

## FUNDING INFORMATION

Funding for the “Black Hole Expedition” to the Sui Shin Hole was received from Caladan Oceanic LLC (USA). Funding for the “Trans‐Pacific Transit Expedition” to the Pacific abyssal plains was received from Inkfish LLC (USA).

## Supporting information


**Video S1.** Abyssal cutthroat eel, *Ilyophis robinsae*, at 4500 m depth beneath the tropical central Pacific Ocean, 0.818°S, 144.303°W (station: 1 TP2_CR6_4500).

**Video S2.** Abyssal cutthroat eel, *Ilyophis robinsae*, at 4484 m depth beneath the tropical central Pacific Ocean, 0.8005°S, 144.295°W (station: 2 TP2_MA6_4500).

**Video S3.** Abyssal cutthroat eel, *Ilyophis robinsae*, at 4573 m depth beneath the Pacific Ocean, 3.9307°S, 144.032°W (station: 3 TP2_OM7_4500).

**Video S4.** Abyssal cutthroat eel, Synaphobranchidae, at 4816 m depth beneath the Pacific Ocean, 6.6435°S, 156.932°W (station: 4 TP3_OM5_4600).

**Video S5.** Abyssal grenadier, *Coryphaenoides yaquinae* at 5568 m depth beneath the central North Pacific Ocean, 32.0665°N, 148.769°W (station: 5 TP1_OM4_5600).

**Video S6.** Abyssal grenadier, *Coryphaenoides yaquinae*, at 4588 m depth beneath the Pacific Ocean, 3.925°SN, 145.883°W (station: 6 TP2_CR70_4500).

**Video S7.** Abyssal grenadier, *Coryphaenoides yaquinae*, at 4937 m depth beneath the Pacific Ocean, 4.4823°N, 145.883°W (station: 7 TP2_MA5_4900).

**Video S8.** Abyssal grenadier, *Coryphaenoides yaquinae*, at 5568 m depth beneath the central North Pacific Ocean, 20.7042°N, 146.272°W (station: 8 TP4_OM2_5400).

**Video S9.** Cusk‐eel, *Bassozetus* sp., at 6328 m depth in the Sui Shin Hole, Philippine basin, 25.142°N, 136.395°E (station: 9 BH_CL4_6300).

**Video S10.** Cusk‐eel, *Bassozetus* sp., at 4588 m depth beneath the Pacific Ocean, 3.925°S, 144.014°W (station: 10 TP2_CR7_4500).

**Video S11.** Cusk‐eel, *Bassozetus* sp., at 4944 m depth beneath the Pacific Ocean, 4.4873°N, 145.866°W (station: 11 TP2_OM5_4900).

**Video S12.** Cusk‐eel, *Bassozetus* sp., at 4465 m depth beneath the Pacific Ocean, 4.6162°S, 144.014°W (station: TP6_CR5_4500).

**Video S13:** Cusk‐eel, *Barathrites iris*, at 4500 m beneath the tropical central Pacific Ocean, 0.818°S, 144.303°W (station: 2 TP2_CR6_4500).

**Video S14.** Cusk‐eel, *Barathrites iris*, at 4500 m beneath the tropical central Pacific Ocean, 0.818°S, 144.303°W (station: 2 TP2_CR6_4500). Note putative feeding event at 4 s.
